# A Suggested Approach of Managing Excessive Maxillary Gingival Display in Terminal Dentition

**DOI:** 10.1155/2020/6975275

**Published:** 2020-11-19

**Authors:** Daniel Saad, Celine Moukarzel, Naim El Haddad, Anthony Rizk

**Affiliations:** ^1^Postgraduate Department, MOI, Faculty of Dentistry, Goethe University, Frankfurt, Germany; ^2^Private Practice, Beirut, Lebanon

## Abstract

The aim of this paper is to report a suggested approach for the management of excessive maxillary gingival display with terminal dentition. A segmental osteotomy of the maxillary process was performed, and the latter used as grafting material for lateral sinus augmentation that was performed simultaneously. Following the graft maturation period, implants were inserted and rehabilitated with a fixed dentogingival prosthesis. Consequently, the mandible was prosthetically restored following the new occlusal plane dictated by the rehabilitated maxilla. Clinically, the procedure showed a drastic improvement in the patient's appearance, eliminating the excessive gingival display. Radiologically, it led to a vertical translation of the maxillary process level in an apical direction. Nevertheless, the resected process used as grafting material was noticed to have a suboptimal behavior as long as it showed increased intrasinusal resorption, barely sufficient for a regular implant accommodation. The described therapy concept seems to be a plausible approach when it comes to manage excessive maxillary gingival displays in edentulous patients or those presenting a terminal dentition. However, at the time of sinus augmentation, authors recommend to graft a mixture of resected maxillary process and a bone substitute material, in order to get more stable results.

## 1. Introduction

Nowadays, social media prevalence interferes with every aspect of one's life. Many bloggers and social media influencers are dictating the way of living by keeping up a stylish lifestyle. Consequently, a person's perfect appearance is becoming a must in society. Hence, dentists are increasingly solicited to maintain their patients' glamourous smiles whereas the business of laminate veneers and smile makeovers is flourishing.

One of many stigmas encountered while smiling is what is commonly known as “gummy smile.” A “gummy smile” refers to an unpleasant excessive maxillary gingival display while smiling. Its clinical correction starts by establishing the right diagnosis in order to find the appropriate treatment. First, an exhaustive facial examination is necessary to determine the facial and lateral proportions and symmetry, as well as the smile line, gum exposure, and lip length. Afterward, an intraoral examination is required for the teeth anatomy, the bite plane, and the periodontal state [1].

Determining the etiology of the excessive gingival display is the next step in the management of such cases. Herein will be presented several diagnoses implicated in the latter.

The first diagnosis is drug and plaque-induced gingival hypertrophy. In this case, gingival inflammation due to plaque or medical treatments—immunosuppressant drugs such as cyclosporine, antiepileptic drugs such as phenytoin, and calcium channels blockers such as nifedipine [2]—can induce a hyperplastic-overgrown gingiva covering the maxillary crowns, hence, causing excessive gingival display. Maintaining impeccable oral hygiene may be the treatment of choice; however, surgical intervention can be indicated in specific cases.

Another mechanism for excessive gum display is originated from an altered passive eruption. In fact, teeth eruption is divided into 2 stages. The first, known as active eruption, consisting of the tooth migration to the occlusal plane, whereas the second stage, known as passive eruption, is due to the migration of the gingiva apically to uncover the tooth. When the passive eruption is altered, the gingiva will still cover the tooth, thus causing shorter clinical crowns and in some cases excessive gingival display. Colset et al. [3] classified the altered passive eruption into two types each having two subtypes:

- Type 1-A: normal distance between cementoenamel junction (CEJ) and alveolar crest with an excessive amount of keratinized tissue and it can be treated by gingivectomy
- Type 1-B: CEJ and alveolar crest at the same level with excessive amount of keratinized tissue, it can be treated by gingivectomy and osseous reduction
- Type 2-A: normal distance between CEJ and alveolar crest with a normal amount of keratinized tissue, it can be treated by apically positioned flap
- Type 2-B: CEJ and alveolar crest at the same level with a normal amount of keratinized tissue, it can be treated by osseous reduction and apically positioned flap

Furthermore, a third cause would be vertical maxillary excess (VME). VME can be defined as a disproportional growth of the maxilla in the vertical direction, which could lead to a long face syndrome [4]. VME is generally associated with an open bite; however, when associated with a normal bite, an excessive gingiva display would hence be covering the incisal edge of the canines [5]. Garber and Salama classified VME into 3 degrees:

- Degree 1: excessive gingival display of 2-4 mm and it can be treated by orthodontic intrusion, crown lengthening, or botulinum toxin injection
- Degree 2: excessive gingival display of 4–8 mm and it can be treated by [6] lip stabilization technique or orthognathic surgery
- Degree 3: excessive gingival display of more than 8 mm and can only be treated by orthognathic surgery

A fourth etiology is a short or hypermobile lip. In fact, short upper lips with less than 15 mm of distance between the lower border of the upper lip and the subnasale [7], or hypermobile lips caused by excessive contractions of the lip elevator muscles, can induce an excessive gingival display. This condition may be treated by the lip stabilization technique [6]. When there is a gingival exposure of 1 to 3 mm, an injection of botulinum toxin is preferred every 6 months to limit the lip elevator activity.

Treatment of excessive gingival display requires a multidisciplinary approach to achieve successful aesthetic and functional results. While the treatment of gummy smile for dentulous patients represents some challenges, albeit more obstacles are encountered with edentulous patients, in order to achieve desirable outcomes.

Complete edentulous upper jaws can be rehabilitated by either conventional removable dentures, or by means of implant-supported prostheses. In the latter, it consists either of a removable implant-supported denture with extended vestibular flanges or a flange-free cross-arch fixed implant-retained prosthesis with (hybrid design) or without (crown design) pink ceramic [8]. Consequently, in order to decide on the optimal therapeutic option regarding the prosthetic design, a diligent complex and multifactorial treatment planning should be considered, which takes into account the volume of the hard and the soft tissue to be compensated, as well as the emergence profile of the future prosthetic teeth along with their vertical, horizontal, and sagittal positions, and provides at once an adequate lip support and balanced facial harmony [8, 9]. Within this scope, a benchmark article [8] addressing this matter was published in 2013 where authors tried to establish a comprehensive decision-making tree with regards to implant-supported prosthesis selection based on the aforementioned patient's clinical characteristics. The published work documented exhaustively different clinical situations of deficient edentulous maxillae. What it did not address is the situations where edentulous maxillae are prominent and excessively visible when smiling.

This paper describes an implant-supported prosthetic rehabilitation of an excessively prominent maxillary process with terminal dentition.

## 2. Case Presentation

**Figure 1:**
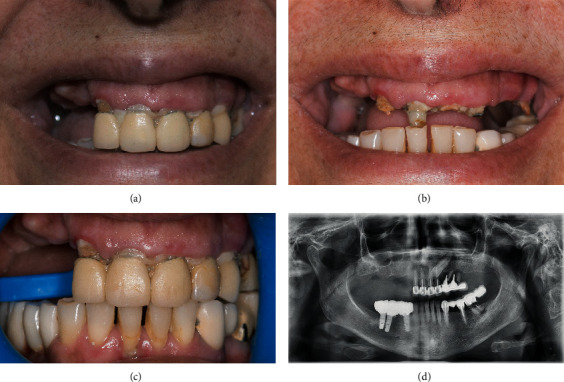
Baseline: (a–c) clinical situation of the patient at the baseline. (d) Radiological situation at the baseline.

In February 2018, one female patient stepped into the private clinic for a complete oral rehabilitation after years of neglected oral care. The patient, who is 58 years old, was seeking for a global dental care and a solution for her excessive gingival display. Intraoral examination revealed a terminal maxillary dentition. Radiographic examination showed an impacted canine #23. In the mandible, apart the present natural anterior teeth, radiologic examination showed the presence of three restored implants on the right side and failed bridge over hopeless teeth on the patient's left side. Extraoral examination revealed a VME reflected by an excessive gingival display at smiling, showing unpleasantly the whole maxillary process all the way up to the tuberosities (Figure 1).

The following treatment plan was agreed on;

Maxilla: extraction of the remaining teeth with alveolar segmental reduction along with a bilateral sinus floor elevation, followed by a maxillary rehabilitation by means of a Toronto prosthesis [10] retained by 6 implants also known as abutment-hybrid overdenture.

Mandible: crowning of the anterior teeth, restoring the left side by implant-supported bridge, and a new set of crowns over the present implants on the right.

**Figure 2:**
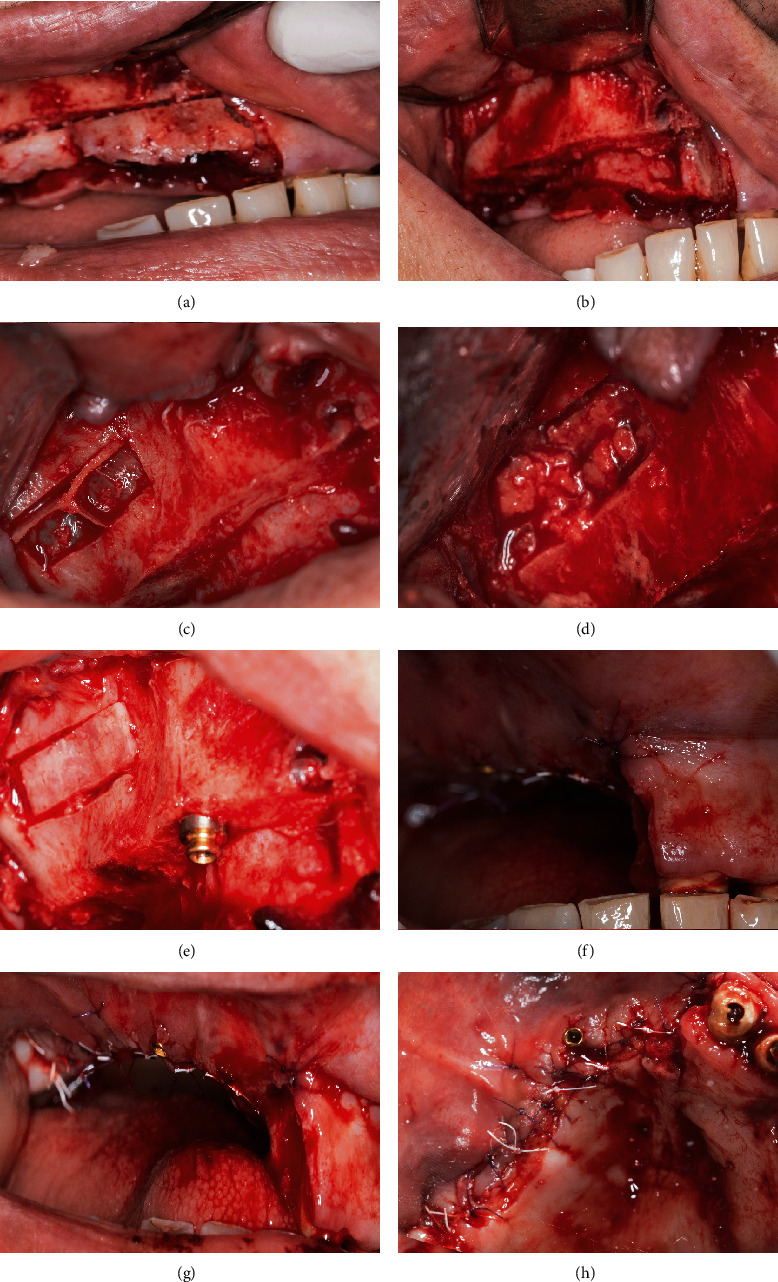
Left hemimaxilla: (a, b) segmental osteotomy of the alveolar process. (c) Lateral window approach during left maxillary sinus floor elevation. (d) Crushed resected alveolar process grafted inside the sinus. (e) Picture showing lateral bony window placed back and implant inserted in the canine region. (f–h) Respectively: frontal, lateral, and occlusal view of the postoperative clinical situation immediately after suturing.

Treatment began by rehabilitating the maxilla:

First, segmental alveolar osteotomy was performed on the right hemimaxilla to remove the vertical maxillary excess using Piezosurgery-touch (Mectron, Cherasco, Italy). The intervention carried out at once a lateral sinus floor elevation, where resected maxillary crest after being crushed, was grafted inside the sinus. Simultaneously, an implant (Duravit 3P, B&B Dental Implant Company, Bologna, Italy) was inserted in the region of the right canine. Then, the buccal flap was repositioned apically to preserve the keratinized mucosa (Figure 2).

**Figure 3:**
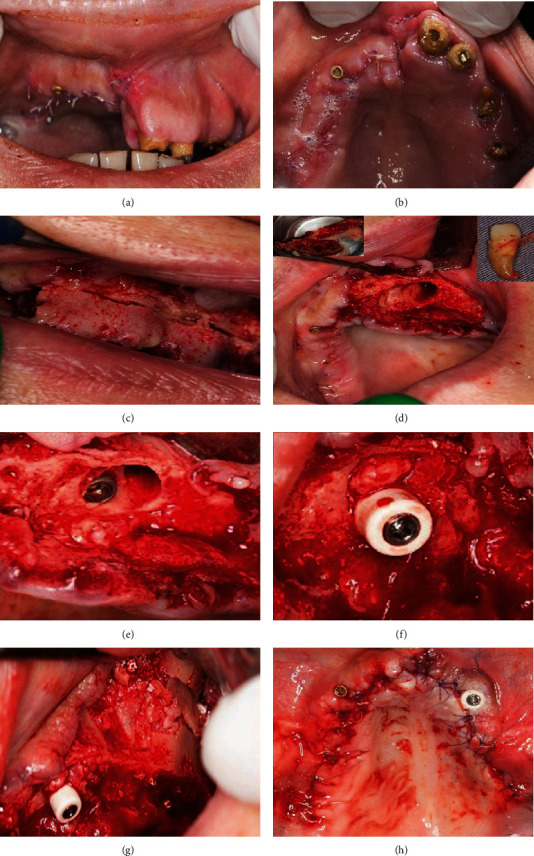
Right hemimaxilla: (a, b) clinical situation 2 weeks following left hemimaxilla resection and immediately before proceeding with the right hemimaxilla. (c) Segmental osteotomy of the right hemimaxilla. (d) Impacted canine extraction. (e) Implant insertion in the remaining canine socket. (f) Jumping space filled with autogenous resected alveolar process. (g) Sinus floor elevation. (h) Occlusal view of the postoperative clinical situation.

Two weeks later, a similar procedure was performed on the left hemimaxilla along with the extraction of the impacted canine. Here, the implant (Duravit EV, B&B Dental Implant Company, Bologna, Italy) was inserted partially in the canine socket, which was filled with the crushed resected alveolar process. The right maxillary subantral space was packed first with bovine xenograft (Ti-Oss, Gyeonggi-do, South Korea) towards the back and then filled with the remaining autogenous resected ridge anteriorly (Figure 3).

**Figure 4:**
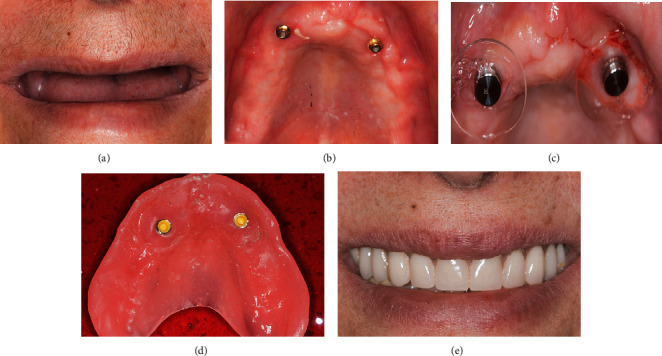
Temporization, 2-3 weeks following right hemimaxilla segmental resection: (a) maximum smile showing the successfully eliminated gingival display. (b) Occlusal view of the healed maxilla with two protruding implant overdenture attachments. (c) Respective housings mounted over the overdenture attachments to be picked-up in the denture intrados by a relining procedure. (d) Denture ready to be seated over the attachments. (e) Patient new smile with the retained denture in the mouth.

After a 2-week healing period, serving as temporary prosthesis during the maturity phase of the grafted sinuses, a removable denture was delivered which was retained by the two inserted implants through their respective transitional implant overdenture attachments (OT equator, Rhein83, Bologna, Italy) (Figure 4).

**Figure 5:**
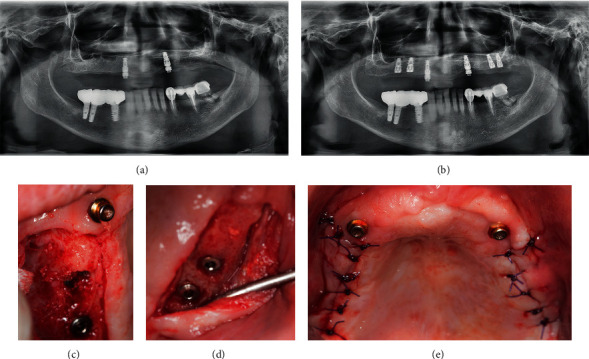
Following sinus graft maturation: (a) Panoramic x-ray 6 months following segmental osteotomy along bilateral sinus augmentation. (b) Panoramic x-ray, 8-months following segmental osteotomy along with bilateral sinus augmentation, and 2 months after implant placement in the sinuses region. (c, d) Two implant placement per each sinus 6-months following bilateral sinus floor elevation. (e) Occlusal view of the maxilla immediately after implant placement and flaps suturing.

6 months later, two implants (Duravit EV, B&B Dental Implant Company, Bologna, Italy) were inserted in each of the sinuses and left submerged for a two-month period (Figure 5).

After 2 months, an open tray impression of the maxilla with its six osseointegrated implants was taken using polyether impression material (Impregum, 3M, Minneapolis, USA), then the poured master cast was scanned with the respective scanbodies in place. Appropriate multiunit abutment was selected for 5 out of the 6 implants, and that for the ease of insertion of the future prosthesis. Consequently, an intermediate Chrome-Cobalt bar was 3D printed using Selective Laser Melting (SLM) technology (SISMA spa, Vicenza, Italy) to check intraorally the passivity fit, which was found to be satisfactory. A diagnostic wax setup using resin denture teeth was then built over the intermediate bar and tried-in intraorally for functional and esthetic evaluation as well as patient approval. The occlusal plane was set parallel to the Camper plane, regardless of the occlusal curve of the mandibular dentition. Then, the diagnostic setup was scanned in-lab, and the final framework of the mesostructure and of its individual crowns was virtually designed.

The mesostructure and the 8 posterior individual crowns' frameworks were 3D printed (SLM) in Chrome-Cobalt alloy, while 6 anterior individual crowns were milled out of zirconia for improved esthetics.

**Figure 6:**
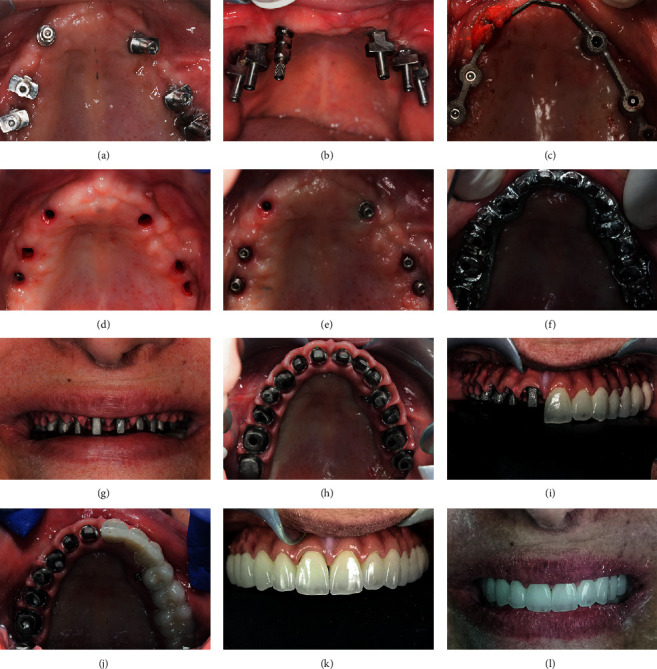
Final rehabilitation of the maxilla: (a, b) Pictures of the impression copings over their respective implants. (c) Passivity fit check using a milled bar. (d) Occlusal view of the maxilla with abutments off. (e) Occlusal view of the maxilla showing five straight multiunit abutments mounted over their respective implants. (f) Try-in of the final milled mesostructure. (g, h) Try-in of the mesostructure after it was layered with pink ceramic. (i, j) Try-in of the individual crowns over the mesostructure. (k, l) Maxillary work completed.

The mesostructure was then manually veneered with gingival pink porcelain (VC gingiva, Creation Willi Geller International GmbH, Meiningen, Austria), while the crowns were built-up with veneering ceramic (Creation Willi Geller International GmbH, Meiningen, Austria), ending up into a hybrid prosthesis. The said prosthesis was screwed-in safely directly over one implant and indirectly through the 5 underlying multiunit abutments, and the individuals crowns cemented on its top subsequently (Figure 6).

**Figure 7:**
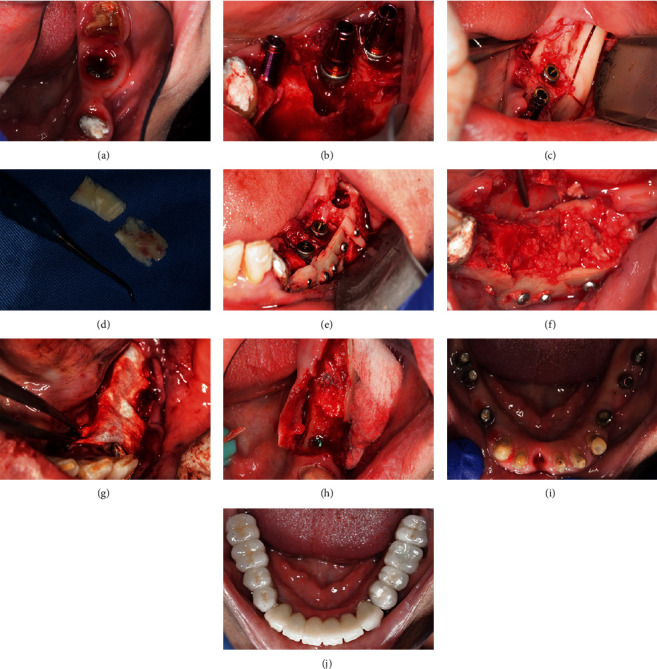
Mandible: (a) Baseline situation of the left hemimandible. (b) Three implants insertion in position #34, #36, and #37. (c) Autogenous bone block harvesting from the external oblique line. (d) Harvested block split in two. (e) Bone block fixation in the recipient site. (f) Autogenous bony particulates filled in the medullary zone. (g) Coverage of the augmentation area by a membrane. (h) Situation at uncovery, 2 months after the augmentation procedure. (i) Abutments and prepared teeth ready to receive their crowns. (j) Mandibular work completed showing the bridges in place.

The mandible underwent a more straightforward treatment plan, which is out of the scope of this report. Concisely, the roots of the teeth #34, #36, and #37 were extracted; then, a flap was raised showing buccal bone deficiency. 3 implants (Rootform, ROOTT, Trate AG, Baech, Switzerland) were inserted along with autogenous horizontal bone augmentation following the principles of the split bone block technique [11]. The bony plates were fixed using titanium microscrews (Conmet LLC , Moscow, Russia). The whole augmentation was then covered by a synthetic resorbable membrane (Tisseos, Biomedical Tissues, Nantes, France). Two months later, implants on both sides were restored prosthetically, with porcelain fused to metal crowns taking into consideration an adequate vertical dimension. The anterior teeth underwent a root canal treatment and received 6 splinted zirconia crowns (Figure 7).

## 3. Results

**Figure 8:**
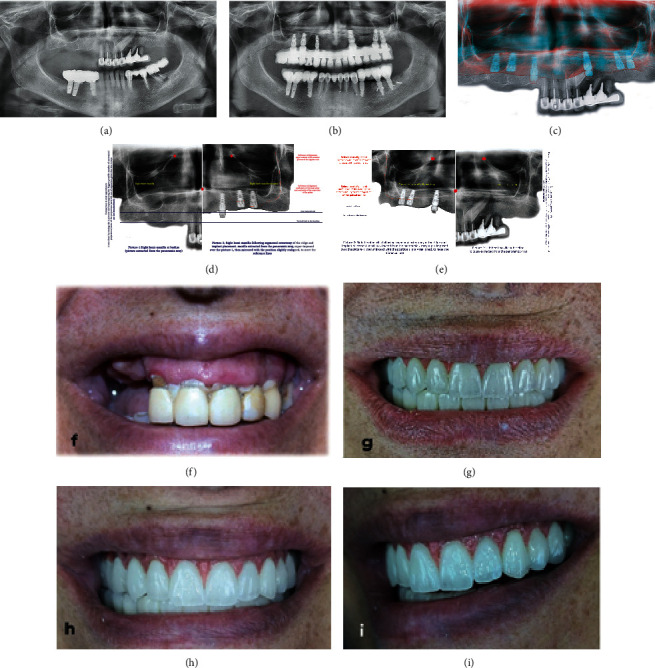
Comparisons. (a, b) Panoramic x-rays at the baseline and after case completion, respectively. (c) 2 overlayed maxillary x-rays, extracted from their respective panoramic (before and after segmental osteotomy) and superimposed to show the approximate difference between the baseline and the actual vertical level of the maxillary process. (d, e) Comparison between the baseline and after osteotomy showing the vertical translation of the maxilla in an upward direction. (f, g) Pictures revealing maximum smile at the baseline and after case completion, respectively. (h, i) Additional pictures showing the final outcome.

Clinically, the procedure showed a drastic improvement in the patient's appearance, eliminating the excessive gingival display. Radiologically, it led to a clear vertical translation of the maxillary process level in an apical direction (Figure 8). Nevertheless, the resected process used as grafting material was noticed to have a suboptimal behavior as long as it showed increased intrasinusal resorption rate, barely sufficient for a regular implant accommodation.

## 4. Discussion

As discussed earlier, little is reported about managing excessive gingival display in edentulous patients. Generally, tooth loss is accompanied by an unavoidable alveolar resorption [9]. This phenomenon, in the posterior region, is usually followed by a sinus pneumatization [12, 13]. Both facts contribute in leaving a minimal posterior ridge height, which impede a proper implant placement in this particular region.

In case of excessive gingival display, when the alveolar vertical reduction is an option, in order to minimize the gummy smile, one may shave off totally the maxillary sinus floors, especially if they are enough pneumatized.

In the present case, and according to their planning, authors reduced the alveolar process and rendered intentionally the case from an excessive gummy smile to a class B situation as per the classification of Avrampou et al. [8], then managed the case accordingly by a fixed hybrid prosthesis.

The rationale behind that was mostly relying on hiding the future mucosal-prosthesis junction away under the upper lip, not to be seen on the maximal smile, as well as giving the required vertical space to fit in the components of the Toronto Bridge, while providing enough room to accommodate a certain height of pink ceramic. On the other hand, ridge reduction was limited by the remaining osseous capital in the anterior region and by the sinuses laterally. Anteriorly, in the canine region, care was taken to leave a vertical height of at least 10 mm in order to insert properly and immediately two regular implants. Posteriorly, since limited by the sinuses, the latter were grafted simultaneously by the resected autogenous material in order to achieve a minimal subantral ridge height able to receive endosseous implants.

Segmental maxillary ostectomy is discussed scarcely in the literature, as a modality of treatment of excessive gingival display [14–16]. Nevertheless, to the extent of the authors' knowledge, the protocol described in this paper is being proposed for the first time: While correcting the gummy smile by segmental osteotomy, the sinus floor elevation is performed concomitally, taking advantage of the resected autogenous alveolar bone that is used as grafting material.

The treatment lasted over a period of a whole year, from osteotomy with sinus floor elevation, throughout the graft maturity, then osseointegration period of the delayed inserted implants, followed by the prosthetic workflow till the final prosthesis delivery, and lastly the mandibular rehabilitation.

Concerning the maxillary prosthetic design, it was opted to rehabilitate the six maxillary implants with a Toronto prosthesis, which consists of a staged fixed prosthesis comprising a screw-retained framework (3D printed (SLM) in this case) that replaces the missing jaw structure. It is designed in a way to be layered with pink material imitating the gum from which emerges a sort of abutment mimicking the prepared teeth as in conventional fixed prosthodontics. As a second stage, come the individual crowns that are intended to be seated over the aforementioned abutments, forming a complex cemented/screw-retained prosthetic construction with the following main advantages:

- Ease of retrievability [10] of the concerned parts in case of maintenance in an event of a repair of chipped ceramic while avoiding compromising the framework, provided that the crowns are cemented with a temporary cement, and the underlying mesostructure is screw-retained
- Achievability of desired esthetics of the individual crowns regardless of the screw access openings situated in the underlying screw-retained framework [17]
- Improved metal framework fitting over the implants, related to the reduced number of repeated porcelain firing over the framework itself, which is responsible for metal shrinkage and a further misfit [18]

Speaking about the drawbacks of the whole procedure:

First, concerning the surgical part, authors agree on the fact that the volume of the obtained bone following sinus floor elevation was hardly sufficient, except the areas where the xenogenic material was inserted, precisely and exclusively in the posterior part of the left maxillary sinus. This clearly reflects the inferiority of the autologous alveolar process grafted into the sinuses in terms of volume stability, an evidence barely reported in the literature [19].

Second, regarding the prosthetic design, despite the aforementioned assets of the abutment-hybrid overdenture, it remains a time-consuming and costly prosthetic option that requires dedication and perseverance of the dental technician, especially when adapting the peripheral porcelain of the prosthetic teeth to that of the underlying screw-retained mesostructure.

At the time of this paper submission, the advent of digital dentistry has been revolutionizing all the aspects of dental medicine. And it is thought that these technologies would have enhanced the proactive planning of this particular case and would have rendered its surgical and prosthetic execution more predictable implying guided bone reductions, and the possibility of instant immediate loading by priorly manufactured provisionals of the guidedly inserted implants, thus significantly shortening the overall treatment period and patient chair time [20, 21].

In conclusion, the described therapy option turns out to be plausible, when it comes to managing an excessive gingival display in a patient with a terminal maxillary dentition. However, assiduous patient selection remains an utmost importance. Also, according to what was witnessed in the present case, regarding the sinus augmentations, the resected alveolar process seems to have suboptimal characteristics as a grafting material. Improvement of the outcome is thought to be achieved by mixing the autogenous alveolar process with a bone substitute biomaterial, to prevent the heavy postoperative intrasinusal graft resorption. In addition, nowadays' unmissable digital tools and 3D printing technologies would increase the predictability of the final outcome.
